# Investigating cognitive-motor effects during slacklining using mobile EEG

**DOI:** 10.3389/fnhum.2024.1382959

**Published:** 2024-05-16

**Authors:** Lara J. Papin, Manik Esche, Joanna E. M. Scanlon, Nadine S. J. Jacobsen, Stefan Debener

**Affiliations:** ^1^Neuropsychology Lab, Department of Psychology, Carl von Ossietzky University of Oldenburg, Oldenburg, Germany; ^2^Oldenburg Branch for Hearing, Speech and Audio Technology (HSA), Fraunhofer Institute for Digital Media Technology (IDMT), Oldenburg, Germany; ^3^Cluster of Excellence Hearing4all, Carl von Ossietzky University of Oldenburg, Oldenburg, Germany; ^4^Center for Neurosensory Science and Systems, Carl von Ossietzky University of Oldenburg, Oldenburg, Germany

**Keywords:** mobile EEG, cognitive-motor interference, dual-tasking, complex balancing, auditory attention

## Abstract

Balancing is a very important skill, supporting many daily life activities. Cognitive-motor interference (CMI) dual-tasking paradigms have been established to identify the cognitive load of complex natural motor tasks, such as running and cycling. Here we used wireless, smartphone-recorded electroencephalography (EEG) and motion sensors while participants were either standing on firm ground or on a slackline, either performing an auditory oddball task (dual-task condition) or no task simultaneously (single-task condition). We expected a reduced amplitude and increased latency of the P3 event-related potential (ERP) component to target sounds for the complex balancing compared to the standing on ground condition, and a further decrease in the dual-task compared to the single-task balancing condition. Further, we expected greater postural sway during slacklining while performing the concurrent auditory attention task. Twenty young, experienced slackliners performed an auditory oddball task, silently counting rare target tones presented in a series of frequently occurring standard tones. Results revealed similar P3 topographies and morphologies during both movement conditions. Contrary to our predictions we observed neither significantly reduced P3 amplitudes, nor significantly increased latencies during slacklining. Unexpectedly, we found greater postural sway during slacklining with no additional task compared to dual-tasking. Further, we found a significant correlation between the participant’s skill level and P3 latency, but not between skill level and P3 amplitude or postural sway. This pattern of results indicates an interference effect for less skilled individuals, whereas individuals with a high skill level may have shown a facilitation effect. Our study adds to the growing field of research demonstrating that ERPs obtained in uncontrolled, daily-life situations can provide meaningful results. We argue that the individual CMI effects on the P3 ERP reflects how demanding the balancing task is for untrained individuals, which draws on limited resources that are otherwise available for auditory attention processing. In future work, the analysis of concurrently recorded motion-sensor signals will help to identify the cognitive demands of motor tasks executed in natural, uncontrolled environments.

## Introduction

Balancing is an everyday life challenge. Human balance control can be described as a feedback system, continually integrating incoming sensory inputs associated with body instability, and inducing corrective actions (Paoletti and Mahadevan, 2012). Individuals may manage this challenge more or less successfully depending on different factors like age, attention, health, fatigue, and physical status. Failing balancing demands usually results in a fall which may lead to severe injury. The risk of falling is even exacerbated when two tasks are executed concurrently, particularly in elderly individuals (Beauchet et al., 2009); whereas children seem to tackle balancing demands better (Schaefer et al., 2008). Distributing our attention between two or more different tasks is, however, very common and important in everyday life (Huxhold et al., 2006). As our population is currently aging, studying the underlying central neural mechanisms of balance control becomes more important (Wittenberg et al., 2017).

One exercise considered a complex balancing task is slacklining (Seidel-Marzi et al., 2021) During slacklining, not only the body’s neuromechanical dynamics but also the external dynamics of the webbing and rope movement introduced by body swaying must be integrated by the feedback system (Paoletti and Mahadevan, 2012). Previous studies found that slackline training enhances postural stability and improves task-related balance performance, (Keller et al., 2012; Pfusterschmied et al., 2013; Giboin et al., 2018) indicating that the feedback system becomes better at integrating sensory stimuli with training. Hence, this whole-body balancing task is not only used as additional training in different sports but also in clinical settings (Seidel-Marzi et al., 2021). Further, practice in complex balancing tasks may prevent falls in older individuals (Sherrington et al., 2011). Studying this sport, especially with the simultaneous performance of an additional task, may consequently contribute to a deeper understanding of the underlying central neural mechanisms of balance control.

The simultaneous performance of two tasks described above is termed dual-tasking (DT). DT can be described as “the concurrent performance of two tasks that can be performed independently, measured separately, and have distinct goals” (McIsaac et al., 2015). During cognitive-motor dual-tasks, i.e., simultaneous execution of a cognitive and a motor task, an effect dubbed *cognitive-motor interference* (CMI) may occur (Al-Yahya et al., 2011). CMI terms a reduction of performance in either one or both tasks, relative to performance in each task individually (single-tasking: ST). It has been argued that the effect is proof of the brain’s limited processing capacity (Plummer et al., 2013). According to the *capacity sharing model*, two tasks sharing neural circuits are both processed when executed at the same time, processing however slows down as the overall task demands exceed the limited capacity (Tombu and Jolicoeur, 2003). Further, the allocation of attentional resources to either one of the tasks plays an important role as the capacity is exceeded (Kok, 2001). However, attentional resources are not allocated randomly but one of the tasks may be prioritized. According to the *posture-first hypothesis*, individuals prioritize the motor task to ensure physical integrity during CMI (Liston et al., 2014; Reiser et al., 2021).

A common neuropsychological method used to assess CMI is electroencephalography (EEG). It has been argued that there is a direct link between the event-related potential (ERP) component P3 and the allocation of cognitive resources and cognitive processing speed in DT paradigms (Kok, 2001). While P3 amplitudes mirror processing capacity (Polich, 1986; Kok, 2001) P3 latencies reflect cognitive processing speed (Yagi et al., 1999). When comparing P3 amplitudes and latencies under DT and ST conditions, amplitudes are often reduced while peak latencies are delayed during DT (Rosenfeld et al., 1993). Hence, the P3 component marks CMI (Kok, 2001) and is therefore frequently used to assess CMI.

The majority of neuroscientific studies in the field have relied on classical laboratory settings, which helped to gain a deeper understanding of the underlying mechanisms. Immobile setups do, however, not allow to investigate DT under real-world conditions. Studies using mobile EEG technology enable the recording of electrical brain activity during natural movements and have become popular over the past 10 years. A robust finding in this field is a reduction of the auditory P3 amplitude during walking as compared to a stationary condition (Debener et al., 2012; De Vos et al., 2014a; Reiser et al., 2019, 2021; Scanlon et al., 2021). Similar results were reported for cycling (Zink et al., 2016; Scanlon et al., 2019) in scenarios where participants could freely move around, but not when a stationary task was performed (Gramann et al., 2010; Zink et al., 2016; Scanlon et al., 2017; Maidan et al., 2019). Regarding latencies, Maidan et al. (2019) reported increased P3 latencies during DT treadmill walking (Maidan et al., 2019). A less frequently studied, but just as important parameter during cognitive-motor DT is the movement itself (Bayot et al., 2018). Studies measuring gait parameters reported that a decline in cognitive performance is accompanied by reduced gait speed or increased gait variability during DT compared to ST gait (Al-Yahya et al., 2011; Patel et al., 2014; Koren et al., 2022). Studies measuring postural sway on a balance pad provide evidence for greater sway during DT compared to ST stance on an unstable surface. These effects are stable across age groups (Ward et al., 2022) but are influenced by cognitive task demands. The more demanding the concurrent task, the greater the postural sway (Pellecchia, 2003). Overall, the findings indicate that DT may decrease auditory P3 amplitudes and prolong P3 latencies in scenarios where participants can freely move. Furthermore, DT can be expected to cause altered gait patterns and increased postural sway.

The current project investigates whether complex balancing and the ability to attentively listen to sounds influence each other in young healthy adults. Participants were asked to perform two tasks simultaneously, while EEG and motion signals were recorded. As a primary task, participants were instructed to stand on a slackline while silently counting targets of an auditory oddball task as a secondary task. Here, participants were instructed to prioritize the oddball. We implemented two control conditions where participants either stood on firm ground while performing the oddball task or stood on a slackline merely, without an additional task. We hypothesize that, compared to standing on firm ground (ST), (i) target-tone induced P3 latencies increase and (ii) P3 amplitudes decrease in the DT condition. Further, we hypothesize that (iii) postural sway increases during DT compared to slacklining without a concurrent task. Since primary task difficulty in the complex balance condition may be lower for more experienced compared to less experienced slackliners, primary task difficulty should be correlated with P3 effects (Polich, 1986). Skilled participants should show a less severe reduction in the P3 amplitude and a weaker peak latency delay during DT compared to less experienced slackliners. It was hence explored whether the change of individual P3 parameters are correlated with skill and if these are mirrored by postural sway.

## Materials and methods

### Participants

Twenty young healthy adults participated in the study [Mean_age_ (*M*) = 27.3 years, standard deviation_age_ (SD) = 4.6 years, 6 female, 14 male]. To be included, participants needed to be able to stand on a slackline for at least 2 min. Participants gave written informed consent and received an honorarium of 10€/h. Due to technical malfunctions during the EEG recording, the data of two participants had to be excluded from further analysis. The final sample consisted of *N* = 18 participants. The experimental procedure was approved by the Ethics Committee of the Carl von Ossietzky University of Oldenburg (Drs.Nr.EK/2021/108).

### Materials

**FIGURE 1:**
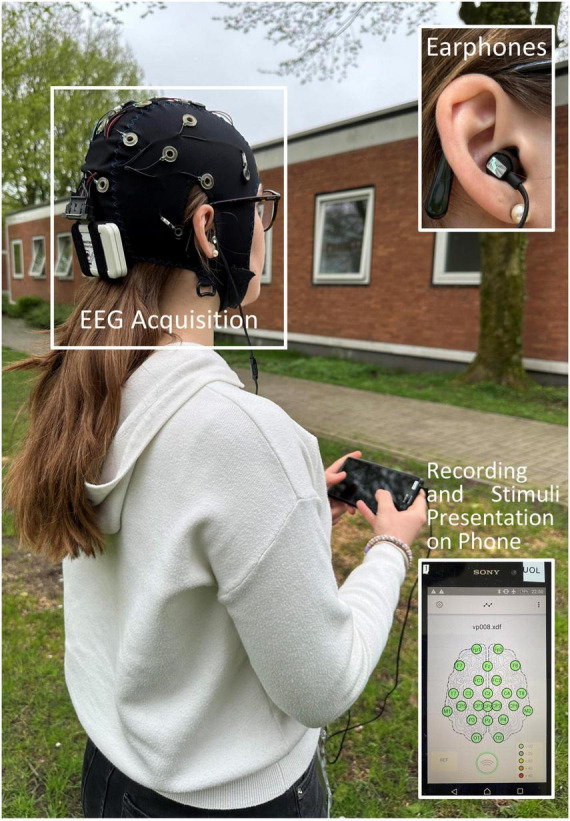
Electroencephalography data were acquired using a 24-channel cap connected to a wireless amplifier. The data were streamed onto a phone via Bluetooth where it was recorded. Stimuli were presented from the phone via earphones. Participants put the phone in a pocket near their waist during the recording.

EEG data were recorded using a wireless amplifier (Smarting, mBrainTrain, Belgrade, Serbia) mounted to the participants’ head capturing 24-channel EEG data using passive wet electrodes (Ag/AgCl sintered) embedded into a custom cap (Easy Cap GmbH, Herrsching, GER). Ground and reference were placed at positions FCz and AFz, respectively. Sampling rate was set to 250 Hz. *Easycap* electrode gel (Abralyt HiCl) as well as light rubbing with a cotton swab were used to lower impedances of all electrodes until a good conductivity (<10 kΩ) was achieved. EEG data were streamed onto a smartphone (Hardware: Sony XPERIA Z1 C6903, Software: Android™ version 5.1.1.) via Bluetooth where it was acquired using the Smarting application (mBrainTrain, version: 4.6) (see Figure 1). Participants placed the smartphone in a pocket near their waist. The Presentation^®^ app (version: 23.0, build 10.27.21 computer, version: 2.1.8. smartphone; Neurobehavioral Systems, Inc., Berkeley, CA, USA) was used to control the experiment. Additionally, gyroscope (Gyro) data were recorded from the amplifier, and accelerometer (Acc) data were recorded from the smartphone. Acceleration [m/s^2^] was sampled with a rate of 250 Hz. The gyroscope measuring angular velocity [°/s] had a sampling rate of 50 Hz. Acceleration, and angular velocity were used as measures of postural sway.

### Procedure

**FIGURE 2:**
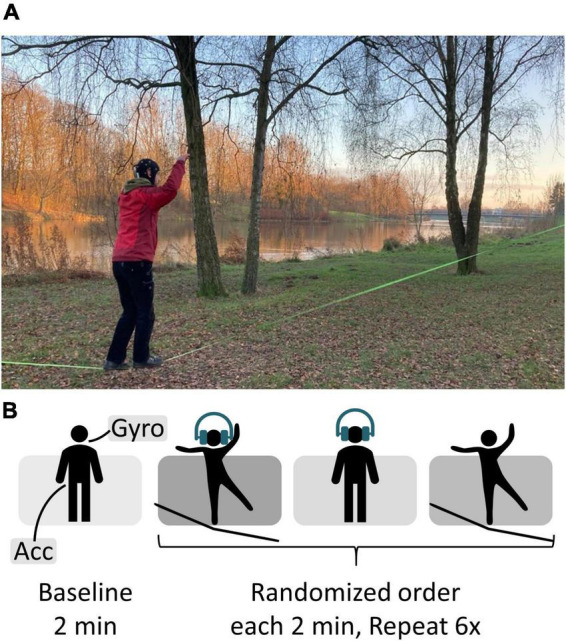
**(A)** Recording setup. At Weserdeich, Bremen, Germany. The participant performs the auditory oddball whist balancing on the slackline (green line, 30 cm above the ground). **(B)** Experimental procedure.

All data were recorded outdoors, mainly at a city park near the city center of Bremen, Germany. First participants performed a 2-min standing baseline followed by an auditory oddball task. It was played binaurally through in-ear headphones (Sony MDR-EX150AP). Volume was individually adjusted according to the participants’ needs, thus the current background noise and their hearing abilities. Overall background noise level was not controlled; however, it was made sure that there were no dominant sound sources nearby. Therefore, the stimulus presentation was not set to a well-defined dB HL range but kept constant throughout the experiment. The participants’ task was to silently count rare target tones presented in a series of frequently occurring standard tones whilst either standing on firm ground or a slackline. The target occurred with a probability of 15%, while 85% of the tones played in the task were non-targets. The two pure tones of the frequency 800 Hz (non-target) and 1,000 Hz (target) had a length of 60 ms. Pre-tone intervals randomly varied between 750 and 1,250 ms with a 125 ms uniform distribution. In addition, participants stood on the slackline without a simultaneous task. The three resulting conditions were split into runs of 2-min length resulting in 18 runs per person, with the order counterbalanced across subjects (see Figure 2). Breaks between runs were self-timed. Participants were given 15 s to get on the slackline before the oddball task started. Per run, between 12 and 18 (15 ± 1.6) target tones were presented. This relatively easy oddball task was chosen to ensure that ERP signal quality could be easily verified even in uncontrolled, mobile conditions. Further specifications of slackline details are provided in Supplementary material. Participants were instructed to immediately step back onto the line if they fell off. They were asked to keep both their arms up whilst standing on the slackline and to fix their gaze to a point at eye level. Participants with corrected vision were instructed to wear glasses.

### Questionnaire data

Participants reported how many deviant tones they counted in a particular run immediately after completing the respective run. This information was noted by the experimenter (see Supplementary Table 4). Participants also filled out a questionnaire asking for demographic data (*Fragebogen zur Ausgangslage*: FAL) prior to EEG data acquisition. Additionally, a short version of the NASA-TLX (National Aeronautics and Space Administration – Task Load Index) was filled out after the recording. This second questionnaire assessed subjective task difficulty by asking to indicate perceived cognitive and physical demands. The questionnaire was used to assess whether slacklining influenced the experienced mental load. To objectively assess the participants’ skill level, they were categorized according to the following criteria. Firstly, the longest longline (min. 30 m long) they ever *send (walk line without falling)* (Balas et al., 2023) and *crossed (walk line with falling)* (Singh et al., 2020) *in meters*. Secondly, the longest highline (min. 35 m above ground) the respective participant ever send and crossed. A highline is usually harder to cross than a longline, since the dynamics of the line as well as other factors (e.g., fear) play an important role. Thirdly, the participant’s current training length in meters (see Supplementary Table 5). In order to evaluate secondary task performance, we calculated how accurately participants counted target tones.

### Data analysis

After acquisition the data were analyzed offline, using MATLAB (© 1984-2020 The MathWorks, Inc., Natick, MA, USA, *Version R2020b 9.9.0*, RRID:SCR_001622), and the MATLAB toolbox EEGLAB (version 2021.0, RRID:SCR_007292) (Delorme and Makeig, 2004). BIDS (RRID:SCR:016124) (Gorgolewski et al., 2016) extended for EEG data (Pernet et al., 2019) was used to organize the data recorded in the current study. After raw data import the channel location coordinates were added. First a 1 Hz zero-phase, non-causal high-pass filter [finite impulse response (FIR) filter, high-pass filter, transition bandwidth: 1 Hz, passband edge: 1 Hz, cut-off frequency: 0.5 Hz, order: 827) followed by a 30 Hz zero-phase, non-causal low-pass filter (FIR filter, low-pass filter, transition bandwidth: 7.5 Hz, passband edge: 30 Hz, cut-off frequency: 33.75 Hz, order: 111) were applied to the data. Then, flatline channels and channels with samples exceeding a threshold of 100 μV or the SD of all channels by 2 SDs were excluded from further analyses. As a result, between 0 and 3 channels (*M* = 1.2 channels) were removed per participant. Subsequently, the datasets were converted into consecutive epochs. Epochs containing atypical artifacts were removed first if they had a joint probability value exceeding 2 SDs (*jointprob*) and second if they had a kurtosis value larger than 2 SDs (*rejkurt*). The resulting data were decomposed using independent component analysis (ICA, *pop_runica, max. 512 steps, extended InfoMax algorithm*). ICA weights were applied to the unfiltered, continuous dataset. *ICLabel* (Pion-Tonachini et al., 2019) was used to identify components representing artifacts. They were removed if their probability of being artifact (Muscle, Eye, Heart, Line Noise, Channel Noise, and Other) related was over 80%. Then a 0.3 Hz zero-phase, non-causal high-pass (FIR filter, high-pass filter, transition bandwidth: 0.3 Hz, passband edge: 0.3 Hz, cut-off frequency: 0.15 Hz, order: 2,751) followed by a 30 Hz zero-phase, non-causal low-pass filter (FIR filter, point low-pass filter, transition bandwidth: 7.5 Hz, passband edge: 30 Hz, cut-off frequency: 33.75 Hz, order: 111) were applied to the data. Epochs ranging from −200 to 800 ms around tone onset were extracted and baseline corrected (−200 to 0 ms). Epochs with atypical artifacts not accounted for by ICA were rejected if their joint probability was exceeded by 3 SDs or if joint kurtosis was exceeded by 3 SDs. This process allowed to retain between 58.9% and 92.5% of trials per participant (*M* = 76.2% ± 9.6%). Data were re-referenced to the average of electrodes Tp9 and Tp10. ERPs were calculated from extracted epochs around stimulus onset. This was done for each movement type (standing/slacklining) and stimulus type (deviant/standard). ERP peak latencies and amplitudes at electrode Pz were determined for each participant and condition by finding the maximum deviation from zero between 200 and 600 ms. Latency was defined as the exact time at which this maximum occurred. To determine the P3 amplitude, a general time window for all participants and conditions was chosen (grand average P3 maximum ±100 ms), respective values were calculated by averaging across this time window. To evaluate ERP noise and data integrity for stationary and mobile recording conditions, we calculated the root mean square (RMS) of each target pre-stimulus baseline (−200 to 0 ms) epoch. We then averaged across all epochs for each movement type and participant, directly following the assumptions of the additive ERP model. This resulted in a measure of EEG noise that could be statistically compared between conditions (De Vos et al., 2014a).

We extracted 2-s segments of Gyro and Acc data simply starting at the beginning of each trial (i.e., 2-min segments the conditions were split into during the experiment). We then calculated SDs across these 2-s segments to assess motion extent (postural sway). This was done for each participant and condition (ST and DT slacklining) individually.

### Statistical analyses

The software RStudio Version 1.4.1106 (© 2009-2021 RStudio, PCB, RRID:SCR_000432) was used to assess the data statistically. Applied packages were *psych, stats, psycho, dplyr, devtools, lsr, DescTools, ggplot2*, and *ggpubr*.

The level of significance for all of the following tests was set to α = 0.05. We first assessed whether RMS, amplitudes, latencies, and accuracies were normally distributed using a Shapiro–Wilk test. Furthermore, the similarity of variances was evaluated by performing an *F*-test of variance on amplitudes and latencies individually. A 2 × 2 repeated measures ANOVA was used to evaluate differences in the amplitudes in different movement type (standing/slacklining) and stimulus type (standard/deviant). Additionally, a one-tailed paired samples *T*-test was calculated on P3 latencies. To correct for multiple comparison of the P3 effects (amplitude and latency) alpha was corrected using a Bonferroni correction (Bonferroni, 1936), resulting in α = 0.025. Finally, Spearman’s rank correlation (Best and Roberts, 1975) was computed to estimate whether the individual change in P3 amplitude and peak latency between standing and slacklining was correlated with participants’ skill level. As RMS and accuracies were not normally distributed, Wilcoxon signed rank tests were calculated to assess whether they differed between types of movement (Wilcoxon, 1945). Effect sizes (Cohen’s *d*/eta squared) were calculated if tests resulted in *p* < 0.05.

To investigate whether motion magnitude during ST and DT slacklining varied, we calculated the SD across the three dimensions *x*, *y*, and *z* for each motion sensor (Acc and Gyro) and averaged these values for consecutive 2 s segments. To obtain a null distribution for the motion data we randomly shuffled the data in time repeatedly (10,000 iterations) and compared the mean SD with the distribution of the permuted data. This process was performed for each participant and each sensor. A binomial test compared the proportion of significant effects of the single participants for both sensor types against the theoretical chance level (50%) proportion. Finally, Spearman’s rank correlation was computed to estimate whether the single subject mean SDs were correlated with participants’ skill levels.

## Results

### Single-trial noise

Distributions of single-trial RMS values were plotted for each participant and movement type individually (see Supplementary Figure 3) Wilcoxon signed rank test revealed a significant difference in RMS between conditions (*V* = 32, *p* = 0.021, *d* = 0.574), with larger RMS during slacklining (slacklining: min. = 0 μV, max. = 8.77 × 10^–5^ μV; standing: min. = 0 μV, max = 5.00 × 10^–5^ μV).

### ERP morphology and topography

**FIGURE 3:**
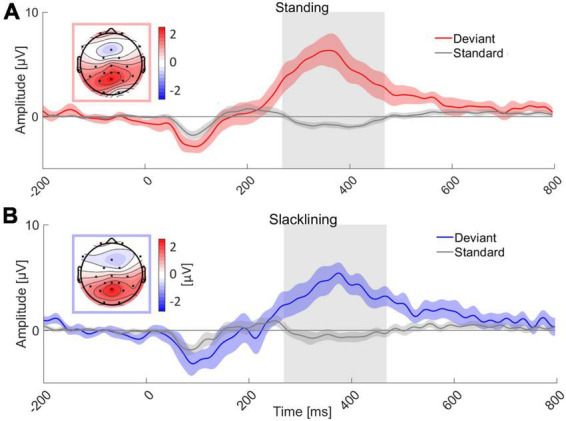
Grand average ERP of all 18 subjects at channel Pz for two movement types; **(A)** standing and **(B)** slacklining. Grand average ERP while standing time-locked to the deviant tone (red) and standard tone (gray) is shown in panel **(A)**. Panel **(B)** depicts similar oddball conditions, these ERPs were recorded whilst participants stood on the slackline (deviant, blue; standard, gray). The topographies represent an average of the amplitudes of all channels measured between 268 and 468 ms either during standing **(A)** or slacklining **(B)**, the respective time interval is marked (gray box) in the graphs. P3 amplitudes are visible in both panels **(A,B)**. The colored areas surrounding ERPs represent the SEM across participants respectively.

Target ERP morphologies appeared to be similar between all conditions, that is, target stimuli evoked a P3 ERP component at electrode Pz, standard stimuli on the other hand did not elicit a P3 (see Figure 3). An auditory evoked potential (N1) was visible in all four conditions. Descriptively, the standard error of the mean (SEM) did not differ between movement types. Topographies were similar in both movement types (standing and slacklining). They showed posterior-central P3 maxima.

### P3 amplitudes and latencies

**FIGURE 4:**
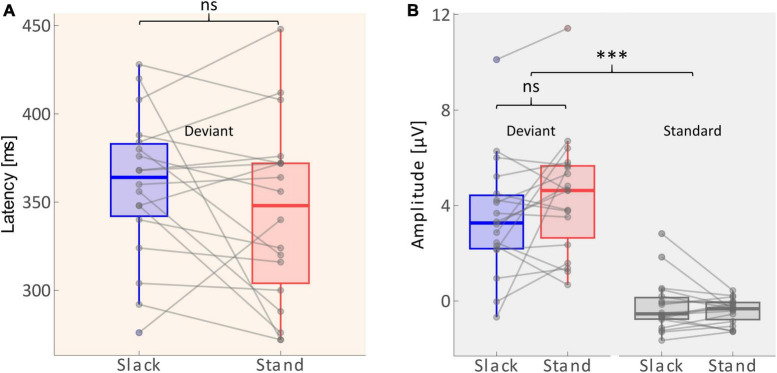
**(A)** Distribution of P3 latencies (between 268 and 468 ms) across sample. Solid lines within the boxes depict the mean. Dots beyond the whiskers represent outliers. Gray lines show individual changes in latencies. **(B)** Distribution of amplitudes (average across 268–468 ms) across the sample (n = 18). The edges of the boxes mark 25th and 75th percentiles. Solid lines within the boxes depict the mean. Dots beyond the whiskers represent outliers. Gray lines show individual changes in amplitudes. Non-significant differences are marked with ns, and significant differences with p < 0.001 are marked using***.

A 2 × 2 repeated measures ANOVA with the factors *Movement Type* (standing/slacklining) and *Stimulus Type* (standard/deviant) was used to evaluate differences in the average amplitudes recorded between 268 and 468 ms after tone onset. The main effect of Stimulus Type was significant [*F*(1,68) = 91.116, *p* < 0.001, η^2^ = 0.573], with larger amplitudes following deviant tones [*T*(35) = 9.769, *p* < 0.001, *d* = 1.628]. The main effect of Movement Type was not significant [*F*(1,68) = 0.616, *p* = 0.435]. Furthermore, there was no significant interaction effect between Stimulus Type and Movement Type [*F*(1,68) = 1.612, *p* = 0.209]. The non-significant effect, however, went into the expected direction, single-subject ERP evaluation shows higher P3 amplitudes in 11 out of 18 participants during standing (see Figure 4B). Amplitudes recorded whilst participants stood on firm ground were about 0.92 μV larger on average. A one-tailed paired *T*-test was performed to test whether the P3 latency was delayed whilst balancing on a slackline compared to standing on firm ground. The test found no significant delay of P3 peak latencies [*T*(17) = −1.362, *p* = 0.095]. Figure 4A depicts that the differences in latencies went into the expected direction. The majority (11 out of 18 participants) had a shorter peak latency during the less demanding movement type. The mean difference across all participants was 16 ms shorter whilst they stood on firm ground compared to slacklining.

To further explore time on task effects we split the runs into first and second-minute segments and analyzed P3 target amplitudes at channel Pz using a 2 × 2 repeated measures ANOVA with the factors movement type (standing/slacklining) and time-on-task (first minute on task/second minute on task). There was no significant effect of time-on-task [*F*(1,68) = 31.477, *p* = 0.059]. Further, there was neither a significant effect of movement type [*F*(1,68) = 16.091, *p* = 0.174], nor a significant interaction between time-on-task and movement type [*F*(1,68) = 0.079, *p* = 0.924].

### Correlations between neurophysiological measures and skill

**FIGURE 5:**
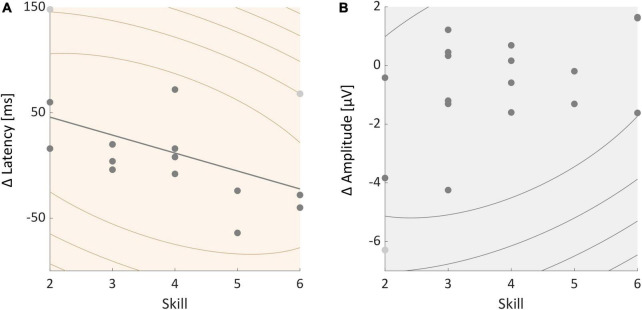
**(A)** Correlation between individual P3 latency effects and participants skill level. **(B)** Correlation between individual P3 amplitude effects and skill. All participants are represented in the graphs, certain dots, however, overlap. Light gray dots represent outliers.

We further tested whether participants’ skill level was correlated with individual P3 amplitude and latency effects. A Spearman’s correlation test found no significant correlation between skill and individual P3 amplitude effects (*rho* = −0.371, *p* = 0.130, see Figure 5B). Skill and P3 latency effects were significantly correlated (*rho* = −0.513, *p* = 0.029, see Figure 5A). The P3 latency was longer during slacklining than standing in less skilled participants. Participants with a higher skill level on the other hand showed a smaller difference or an even shorter latency in the slackline condition compared to standing.

### Movement data

**FIGURE 6:**
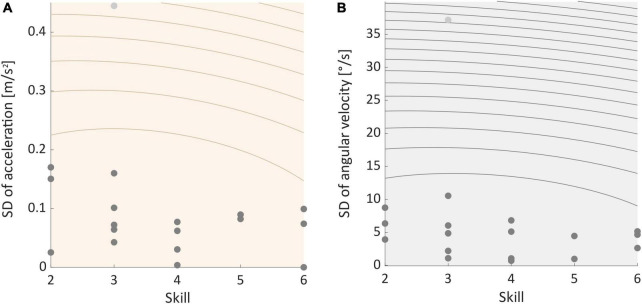
**(A)** Correlation between individual standard deviation of acceleration and participants skill level. **(B)** Correlation between individual standard deviation of angular velocity and skill. Light gray dots represent outliers.

Two-sided permutation tests indicated that SDs of movement data recorded during ST- and DT-slacklining did significantly differ in 14 (acceleration) and 13 (angular velocity) participants, respectively (see Supplementary Tables 1–3). The proportion of significant findings in the permutation tests was significantly above chance level for both sensor types (Acc data: 14 of 18, *p* = 0.015; Gyro data: 13 of 18, *p* = 0.048). *Post-hoc* one-sided permutation tests revealed that SD was greater in the ST slacklining – compared to the DT condition. Binomial tests confirmed that the proportion of significant findings was significantly above chance level for accelerometer data (14 of 18, *p* = 0.015) but not for gyroscope data (11 of 18, *p* = 0.240). Further, the pattern of results we found did not correlate with participants’ skill level, which was the case for both sensor types (Acc: *rho* = 0.165, *p* = 0.513, see Figure 6A; Gyro: *rho* = 0.120, *p* = 0.635, see Figure 6B).

### Questionnaire results

The NASA-TLX data of four participants were missing due to human error. They were hence excluded from statistical analyses. Participants who answered the questionnaire rated the mental demand (8.0 ± 4.8), physical demand (9.3 ± 4.5), and effort (9.9 ± 4.7) as medium. Temporal demand (4.7 ± 3.9), and frustration (5.2 ± 4.8) were rated as medium to low. Performance (3.9 ± 4.3) was rated as good. All categories, however, showed a large standard deviation across participants. A non-parametric one-sided Wilcoxon signed rank test was performed to assess whether participants counted target tones more accurately whilst standing on firm ground. Accuracies did not differ significantly between movement types (*V* = 74, *p* = 0.632). The percentage of correctly counted target tones in both movement types was very high (slacklining: 97.2% ± 3.9%, standing: 97.5% ± 3.5%).

## Discussion

We here demonstrated that good-quality ERPs can be obtained from EEG recordings taken during a complex balancing task, in a natural outdoor environment. ERPs morphologies and topographies were similar in stationary and balancing conditions. Consistent with previous studies (Ladouce et al., 2017; Scanlon et al., 2017, 2019; Reiser et al., 2019) topographic analysis confirmed that the P3 was most prominent at posterior scalp site, suggesting a P3b potential (Conroy and Polich, 2007).

### P3 effects

As expected, a significant main effect of stimulus type on amplitude was found in the P3 time-window of interest, showing that the experimental manipulation was effective. These results echo numerous previous studies, showing the neural response to deviant sounds (Polich, 2007; Debener et al., 2012; Scanlon et al., 2022). Interestingly, contrary to our hypothesis, the type of movement had no significant effect on target elicited P3 amplitudes. Previous studies have found medium to large effects on P3 amplitudes while free walking (Debener et al., 2012; De Vos et al., 2014a,b; Reiser et al., 2019, 2021; Scanlon et al., 2022), and cycling (Scanlon et al., 2019), while no significant effect of stationary exercise on P3 has been found in others (Zink et al., 2016; Scanlon et al., 2017). Ladouce et al. (2019) described larger P3 amplitudes during treadmill compared to hallway walking (stationary vs. freely moving), and static vs. dynamic view, they conclude that free movement is more demanding due to increased visual and inertial demands (Ladouce et al., 2019). A possible explanation for the absence of a significant effect observed in the current study could hence be the stationary scenario with a lack of visual flow. Additionally, the run length of 2 min was rather short. While previous studies have used substantially longer silent counting oddball run durations (e.g., Debener et al., 2012, 2015), balancing on a slackline much longer than 2 min would have been a real challenge even for the experienced slackliners that participated in this study. As a result, the shorter run length has probably led to a rather low cognitive load of the oddball task demands. Taken together this may have resulted in a lower task difficulty than in other studies. The absence of P3 amplitude effects on group average marks similar cognitive demands during the slackline and the standing condition. Here, we did not observe a shift of shared cognitive resources toward the complex balancing task (Polich, 1986; Kok, 2001). In hindsight, sitting might have been a more suitable control condition.

We hypothesized that P3 latencies would increase during slacklining compared to standing. Yet, P3 latencies were not modulated by movement type. A previous study reported similar latencies in both sitting and stationary cycling (Zink et al., 2016). Another study found longer P3 latencies during walking compared to standing (Maidan et al., 2019). The components’ peak latency is thought to be delayed as primary task difficulty increases (Polich, 2007). Therefore, the difference in primary task difficulty between standing and slacklining could have been too small to yield detectable effects on the cognitive processing speed reflected by the P3 latencies in trained slackliners (Yagi et al., 1999).

Although single-trial noise significantly differed between conditions, ERP amplitudes measured following standard tones did not differ between movement types. Specifically, the N1 morphology was similar in both movement types. It is therefore rather unlikely that noise level differences are responsible for the P3 amplitude reduction. Scanlon et al. (2019) reported a higher level of noise whilst cycling compared to sitting, and walking can induce movement artifacts in mobile EEG recordings (Jacobsen et al., 2021). While Scanlon et al. (2019) did not report on the effect size, they concluded that CMI, rather than the difference in noise was responsible for the effect of task on the P3.

Regarding individual differences, for P3 latency but not amplitude we found an interference effect for less skilled individuals, whereas individuals with a high skill level may have even shown a facilitation effect. This result indicates that participants with a high skill level most likely experienced a small if any, difference in primary task difficulty between the movement types. These results mirror the findings of different studies that demonstrated that experts usually outperform novices when asked to maintain their performance in dual-tasking scenarios (Schaefer, 2014). Other researchers reported on cognitive-motor facilitation during moderate stationary exercise, such as treadmill walking (Penati et al., 2020; Patelaki et al., 2023). The same individuals showed CMI effects during more demanding tasks like overground walking (Penati et al., 2020). These studies argue that prefrontal resources accompanied by increased arousal levels and improved task performance may be more adequately activated by moderate exercise (Patelaki et al., 2023). Such activations may lead to facilitation effects. We assume that the definition of moderate exercise is highly individual depending on many factors such as training hours and effort one puts into a specific task. It is hence likely that skilled individuals experienced the task effort as moderate while less skilled individuals experienced a high motor task load. We argue that these differences are reflected by either interference or facilitation effects indicated by individual P3 effects. Hence, not only factors like primary task load, age, anatomical differences (Conroy and Polich, 2007; Yerlikaya et al., 2022), or disease (Polich, 2007) play a role. We conclude that individual motor skill differences influence how strong individual P3 dual-tasking effects are.

### Movement differences

Movement patterns, that is postural sway, differed significantly between conditions. Yet, neither the change in acceleration nor the change in angular velocity correlated with participants’ skill levels. Contrary to our hypothesis we found more acceleration in the single-tasking condition while angular velocity did not differ significantly between conditions. Higher acceleration values may indicate a less stable stance on the slackline. This interpretation is in line with a previous study by Huxhold et al. (2006). Here, postural control performance increased when participants performed easy cognitive tasks while standing on a force platform compared to standing on the platform without an additional task. This effect was reversed when cognitive tasks were more difficult (Huxhold et al., 2006). The platform they used was stable and therefore may not represent a complex balancing task very well. Nevertheless, an explanation for the deviation of our results from other literature (Pellecchia, 2003; Ward et al., 2022) could be the cognitive task used since an oddball task does not pose strong cognitive task demands.

Beilock et al. (2002, 2004) found that expert golfers and soccer players perform better under DT conditions than when purely focusing on the skill, thus ST, this pattern was reversed in novices. As with golf and soccer, active attention allocation toward slacklining may have had a negative impact on performance in the ST condition, as none of our participants were beginners. Another possible explanation for these unexpected results could be the Hawthorne effect. According to the effect, participants change their natural behavior because they know they are under observation while participating in a study (McCarney et al., 2007). The effect has previously been reported to alter gait characteristics during treadmill walking (covert vs. overt evaluation) using a motion capture system (Farhan et al., 2023). Furthermore, Andersson et al. (2002) provided evidence that postural sway becomes greater when participants are instructed to rate their sway, thus, focus on their balance (Andersson et al., 2002). Participants likely felt more observed and consequently tended to focus on their postural sway in the absence of distraction through the cognitive task. Their behavior might have been altered through these factors, resulting in a less stable stance in the ST condition.

Further, movement differences to study using motion sensors would have been the characteristics and frequency of fall occurrences as well as their relation to skill level and condition (ST vs. DT). However, none of the participants included in the statistical analysis fell off the slackline during recording. Unfortunately, one participant fell off during an early run, due to personal reasons the experiment was terminated and not enough data from this person could be acquired. Besides, the posture first hypothesis (Liston et al., 2014) could explain our findings. The hypothesis states that resources in dual-tasking are not allocated randomly, but priority will likely be on motor tasks to ensure physical integrity (Liston et al., 2014). Participants likely prioritized their posture during slacklining in the dual task condition to avoid falls. Movement patterns therefore represent better (acceleration) or similar (angular velocity) posture during DT. Taken together with the neurophysiological results we provide evidence that the paradigm led to a modulation of cognitive performance while motor performance seemed to be facilitated likely because of an interaction of rather low cognitive task demands with prioritized posture and focus on posture control during DT.

### Questionnaire data

Participants rated their performance as good and the overall task demands, effort, and frustration across the experiment as low to medium. Participants in previous cognitive-motor interference studies rated task demands higher during more complex movement types (Reiser et al., 2019). A collection of behavioral data (NASA-TLX) for both movement types individually could improve the assessment of task demands. This would further contribute to evaluating primary task difficulty and its relationship to the factor skill.

In the current project, accuracies did not differ significantly between types of movement. These results are in line with a previous study assessing CMI whilst skateboarding (Robles et al., 2021). Our findings, however, deviate from other findings where accuracy was found to be reduced as movement complexity increased (Reiser et al., 2019, 2021). Furthermore, participants counted targets less accurately in previous studies. One important difference between the current- and other studies assessing CMI using a similar cognitive task (Scanlon et al., 2017, 2019; Reiser et al., 2019, 2021), was that the time participants had to execute the task was substantially shorter here, participants needed to sustain attention for a shorter period of time. This might have influenced the accuracies positively. The average accuracy in both standing and slacklining was over 95%. It can thus not be excluded that ceiling effects influenced the outcome. Moreover, the computation of accuracies in this study was based on participants’ reports on the number of target tones they counted within one trial. It was hence, impossible to distinguish between correctly counted targets and false positives. Authors of previous publications asked participants to press a button whenever they heard a target tone (Reiser et al., 2019, 2021). This approach could have improved accuracy acquisition in the current study. Here participants, however used their arms to tackle the balancing demands posed on them through slacklining, a button in one of their hands would likely have influenced balancing performance.

### Future directions

The effect of primary task difficulty on the P3 of an auditory oddball is well-known and has been replicated many times using different movement types. Assessing the effect of skill on an auditory P3, however, is a novel approach. It would, hence, be interesting to evaluate the effects of skill on the P3 during different types of movement including a greater variety of skill levels, i.e., novices and professionals. Exploring the impact of visual flow, run length and choice of control conditions on P3 effects is crucial. This could be implemented by adding conditions to the already existing paradigm, one such condition could introduce a visual flow to the stationary setup, while another could extend the duration of runs (e.g., until participants are no longer able to maintain balance on the slackline). Further, when assessing CMI in real-life scenarios brain, movement data, and other factors such as the Hawthorne effect on measures should be taken into consideration.

### Limitations

We aspired to keep all factors constant between conditions. While out-of-the-lab studies may suffer from a lack of standardization, the replication of laboratory findings in real-world environments can help to identify robust patterns of results. It is unlikely that the magnitude of environmental distraction (e.g., street noise, passing by passengers) posed a systematic confound. However, to evaluate their influence, future studies should consider monitoring these context factors (Holleman et al., 2020). Another limiting factor is our relatively small sample size since we could include only those as participants who could easily balance on a slackline for several minutes. This requirement minimized our chances of acquiring data from a large sample size, despite strong efforts to include suitable candidates from the north-western region of Germany.

## Conclusion

The present study adds to the growing field of CMI research in naturalistic environments. Contrary to prior work we found no CMI effects marked by P3 effects (Debener et al., 2012; De Vos et al., 2014a; Ladouce et al., 2017; Reiser et al., 2019; Scanlon et al., 2019; Liebherr et al., 2021). Lower task load through the stationary scenario and comparatively short condition length may explain these results. Moreover, we suggest that the factor skill, which correlated with individual P3 latency but not amplitude, interfered with the outcome. We argue that the individual CMI effects on the P3 ERP reflects how demanding the balancing task is for untrained individuals, which draws on limited resources that are otherwise available for auditory attention processing. This interference has most likely also contributed to the rather unexpected results. Regarding movement patterns we provide deviating results from previous literature where postural sway was found to be increased during DT- compared to ST balancing (Pellecchia, 2003; Ward et al., 2022). Again, low cognitive task demand through short trial duration and a rather easy task could explain these results (Huxhold et al., 2006). Besides, awareness of observation through the experimenter leading to increased focus on posture during ST and prioritization of posture during DT may have led to better posture control and stability during DT. Taken together, we demonstrate the feasibility of mobile EEG acquisition during human balance control and provide evidence that cognitive and motor processes may draw from the same pool of processing resources that may be misleadingly labeled as purely cognitive.

## Data availability statement

The raw data supporting the conclusions of this article will be made available upon request by the authors, without undue reservation.

## Ethics statement

The study was reviewed and approved by the Ethics Committee of the Carl von Ossietzky University of Oldenburg Q20 (Drs.Nr.EK/2021/108), Oldenburg, Germany. The studies were conducted in accordance with the local legislation and institutional requirements. Written informed consent was obtained from the individual(s) for the publication of any identifiable images or data included in this article.

## Author contributions

LP: Conceptualization, Data curation, Formal analysis, Funding acquisition, Investigation, Methodology, Project administration, Visualization, Writing – original draft, Writing – review & editing. ME: Conceptualization, Data curation, Funding acquisition, Methodology, Writing – original draft, Writing – review & editing. JS: Conceptualization, Methodology, Supervision, Writing – original draft, Writing – review & editing. NJ: Conceptualization, Methodology, Supervision, Writing – original draft, Writing – review & editing. SD: Conceptualization, Funding acquisition, Methodology, Resources, Supervision, Writing – original draft, Writing – review & editing.
