# Supplementary material for: Investigating cognitive-motor effects during slacklining using mobile EEG
Source: Front Hum Neurosci. 2024 May 16;18:1382959. doi: 10.3389/fnhum.2024.1382959 (PMC11137308; doi:10.3389/fnhum.2024.1382959)

Supplementary Material

# Supplementary Data

## Slackline Specifications

Slackliners (like other athletes) tend to get used to the kind of slackline they use the most. A line that has become easy for a beginner (e.g., 10m strongly tensioned line), can be very challenging for an advanced or even professional slackliner since experienced slackliners usually walk longer lines which behave differently form shorter lines. During the experiment the slackline was tensioned between two trees at 1.8m. It had a length of 19m, and a maximum distance of 0.3 m from the ground at the place where subjects stood. An Equilibirum Ò slim webbing, with a maximum working load (WLL) of 3 kN, a minimum breaking load (MBS) of 11 kN, a weight of 26 g/m, a webbing width of 25 mm, and a stretch of 7% (při WLL) was used for the experiment (https://www.eqb.cz/slim-en.html). It was tensioned using the so called Primitive Tensioning System. This setup was chosen for its balance between being close enough to the behaviour of a very long line while still being not too difficult for an intermediate slackliner to balance on. That way, it was possible to use the same line to assess highly skilled - and intermediate skilled slackliners.

# Supplementary Figures and Tables

## Electrophysiological Data

Supplementary Figure 1 depicts preprocessed event related potentials (ERPs) split into movement type and stimulus type for each participant individually. Grand average ERPs are shown in Figure 3. Grand average P3 topographies are shown in Supplementary Figure 2 A and B, topographies of non-target trails for the same time respectively are shown in Supplementary Figure 2 C and D.


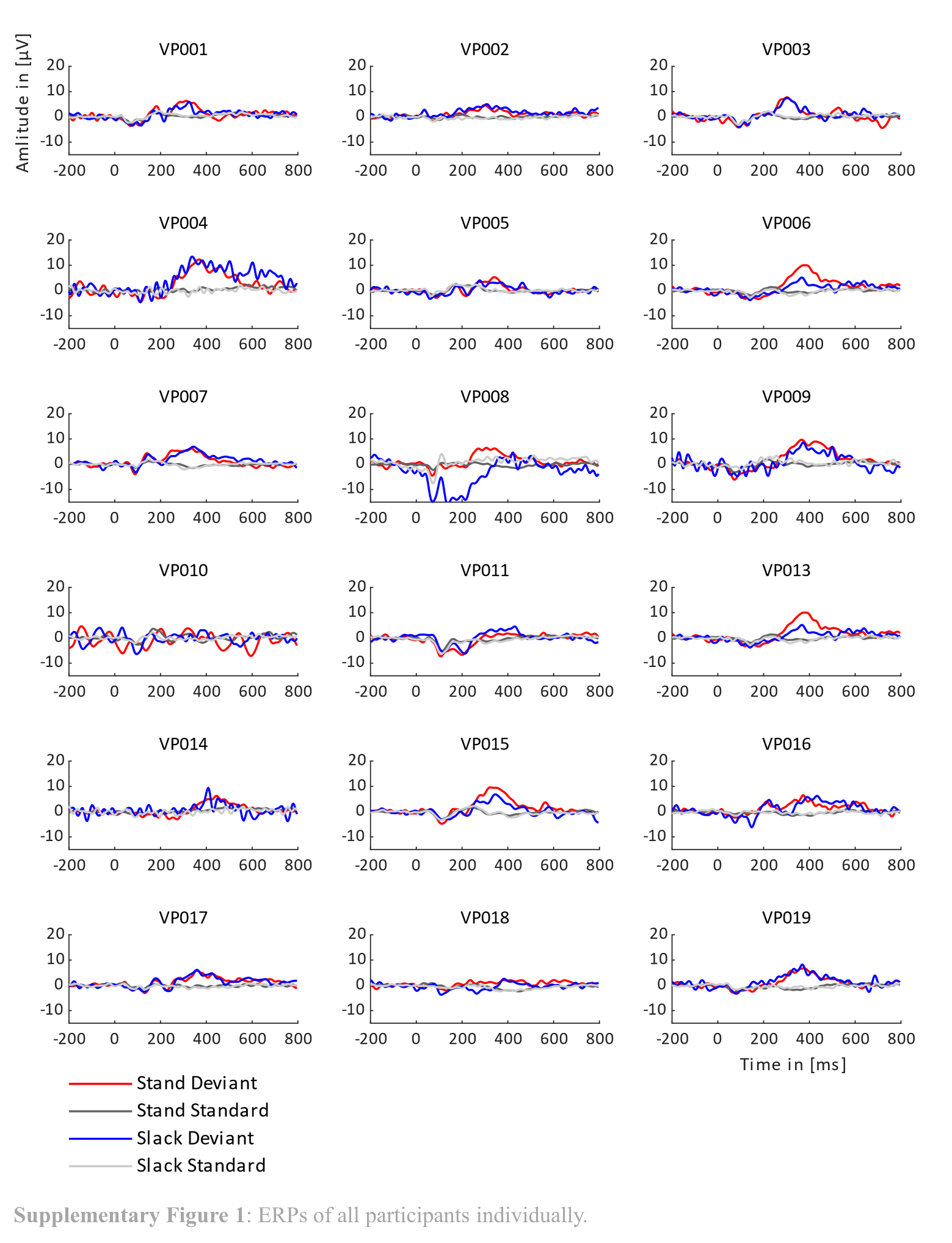


.
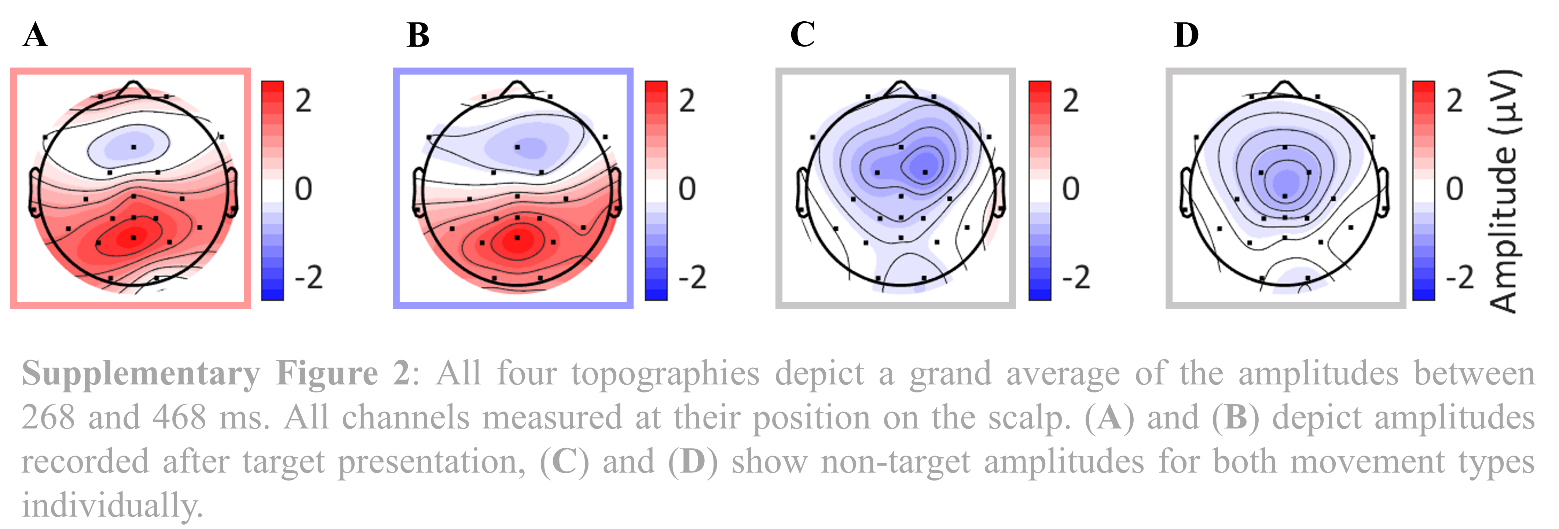


To assess the ERP noise and ensure data integrity under mobile recording conditions, we computed the root mean square (RMS) of the pre-stimulus baseline (-200 to 0 ms) for each participant separately (see Supplementary Figure 3). Subsequently, we averaged across all epochs for each movement type and participant. Using this approach we obtained a measure allowing statistical comparison between movement conditions.

## Movement Data

To investigate if there were differences in motion magnitude during single-task (ST) and dual-task (DT) slacklining, we first calculated the standard deviation (SD) across the three dimensions (x, y, and z) for each motion sensor (accelerometer and gyroscope). We then averaged these SD values over consecutive 2-second segments. To establish a baseline for comparison, we randomly shuffled the motion data in time 10,000 times and compared the mean SD with this permuted data distribution. This process was repeated for each participant and each sensor. A visualization of these distributions for each participant and sensor type can be seen in Supplementary Figure 4 for acceleration and 5 for angular velocity (gyroscope).


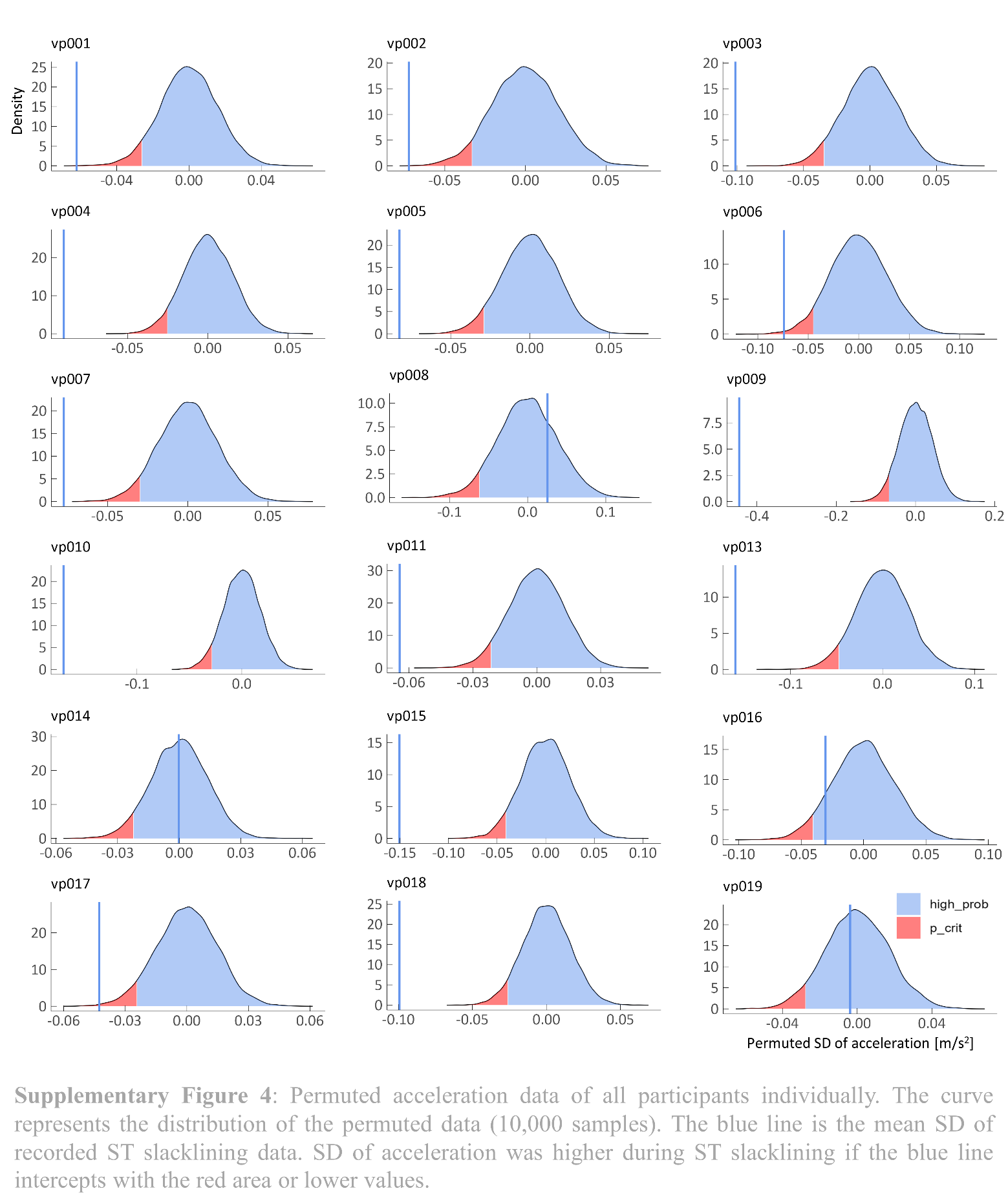


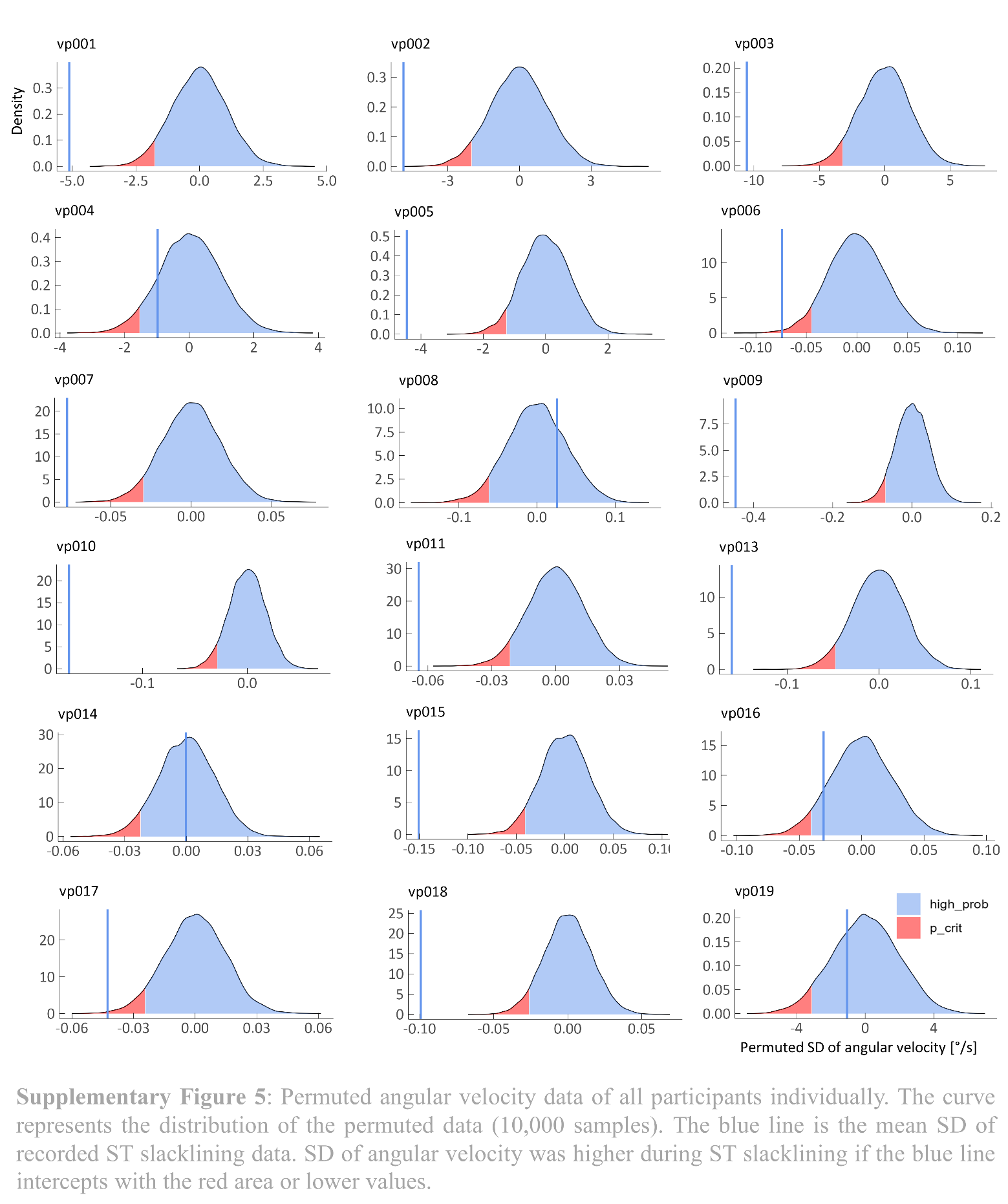


Using a binomial test, we assessed whether the proportion of significant effects observed in individual participants for both sensor types differed from the theoretical chance level of 50%. Two-sided permutation tests revealed significant differences in SDs of movement data recorded during ST and DT slacklining in 14 participants for acceleration and 13 participants for angular velocity, respectively (see Supplementary Table 1). Post-hoc one-sided permutation tests revealed that SD was not greater in the DT slacklining compared to ST (see Supplementary Table 2). However, according to the tests SD was greater in the ST slacklining compared to the DT condition. Binomial tests confirmed that the proportion of significant findings was significantly above chance level for accelerometer data (14 of 18, *p* = 0.015) but not for gyroscope data (11 of 18, *p* = 0.240, see Supplementary Table 3).

**Supplementary Table 1**: Permutation test; two sided


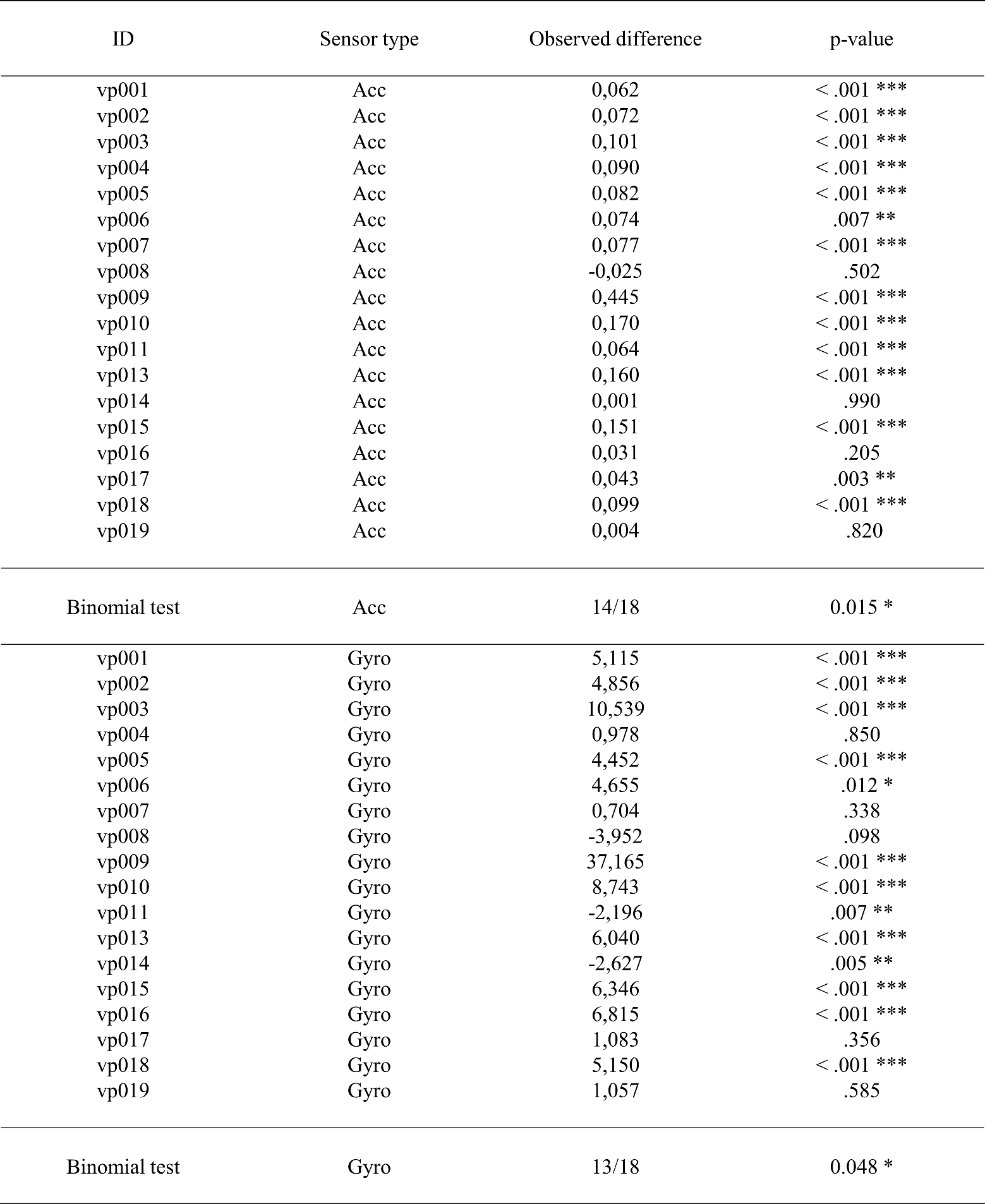


**Supplementary Table 2**: Permutation test; One sided (Slack DT< Slack ST)


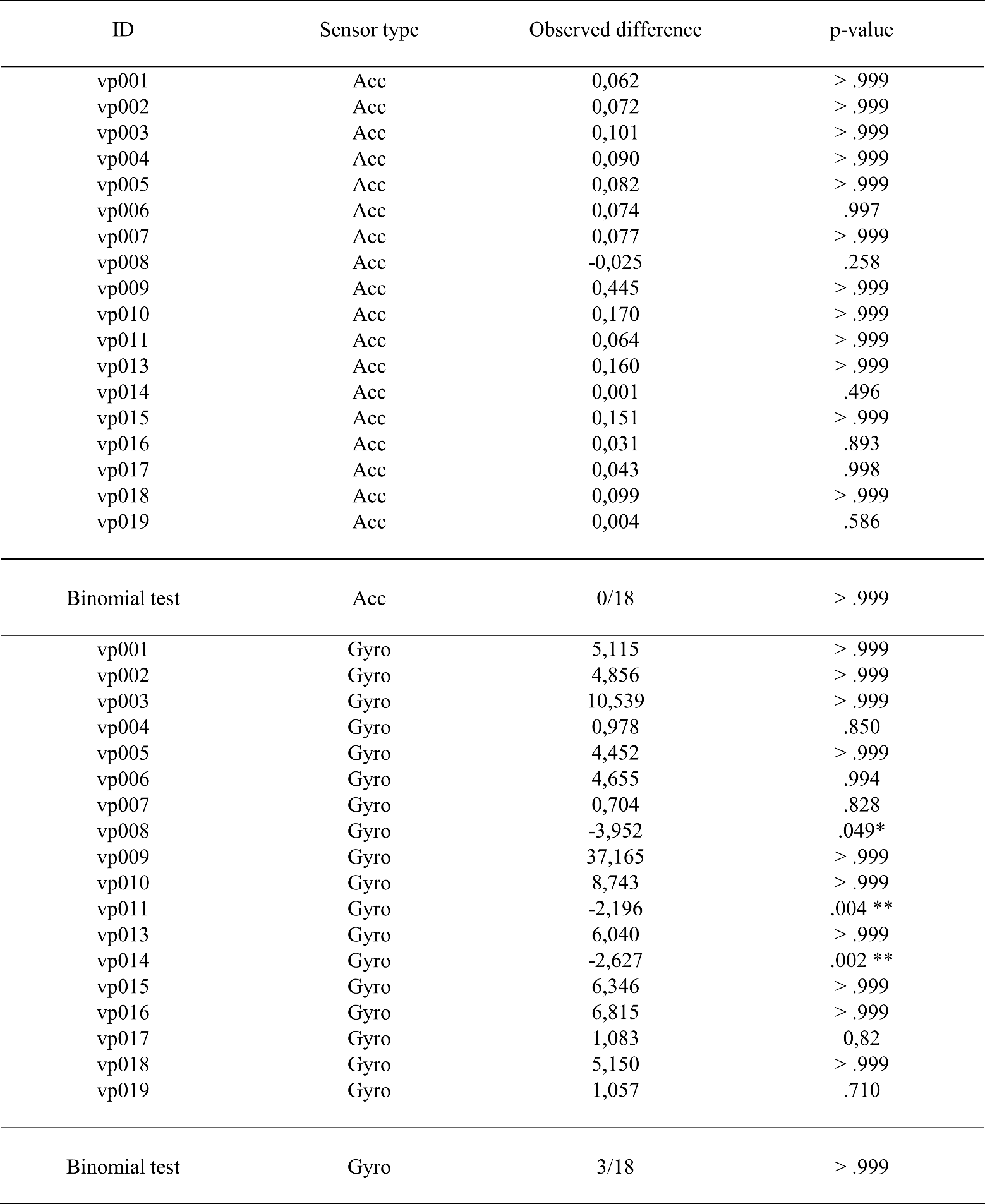


**Supplementary Table 3**: Permutation test; One sided (Slack DT > Slack ST)


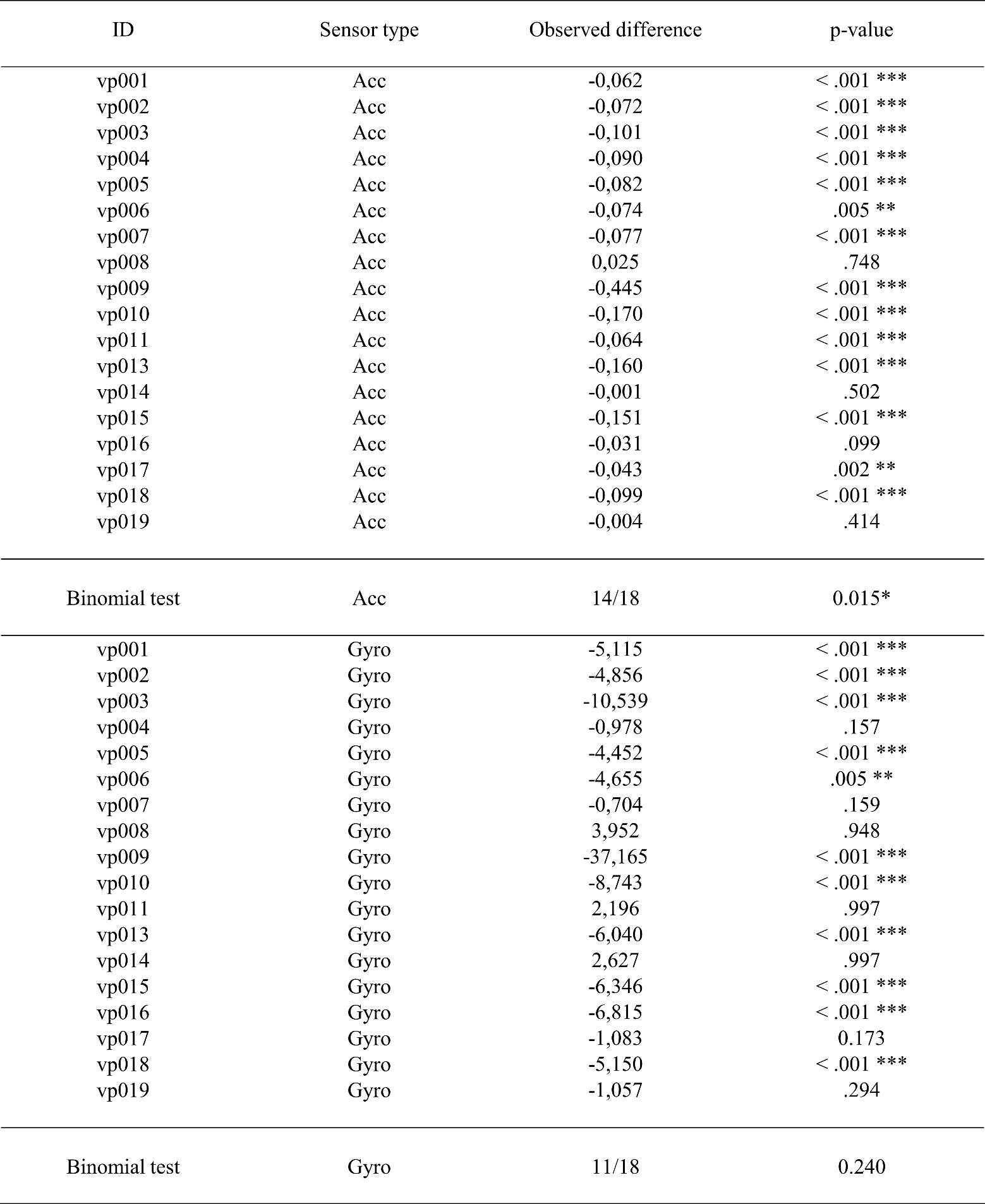


## Questionnaire Data

Participants provided feedback on the number of target tones they perceived and counted in a specific run immediately after completing the corresponding run. The experimenter noted this information on a questionnaire (see Supplementary Table 4). This resulted in three different counts per trial, since each trial had three runs, and 18 counts per participants.

**Supplementary Table 4**: Questionnaire for deviant counts


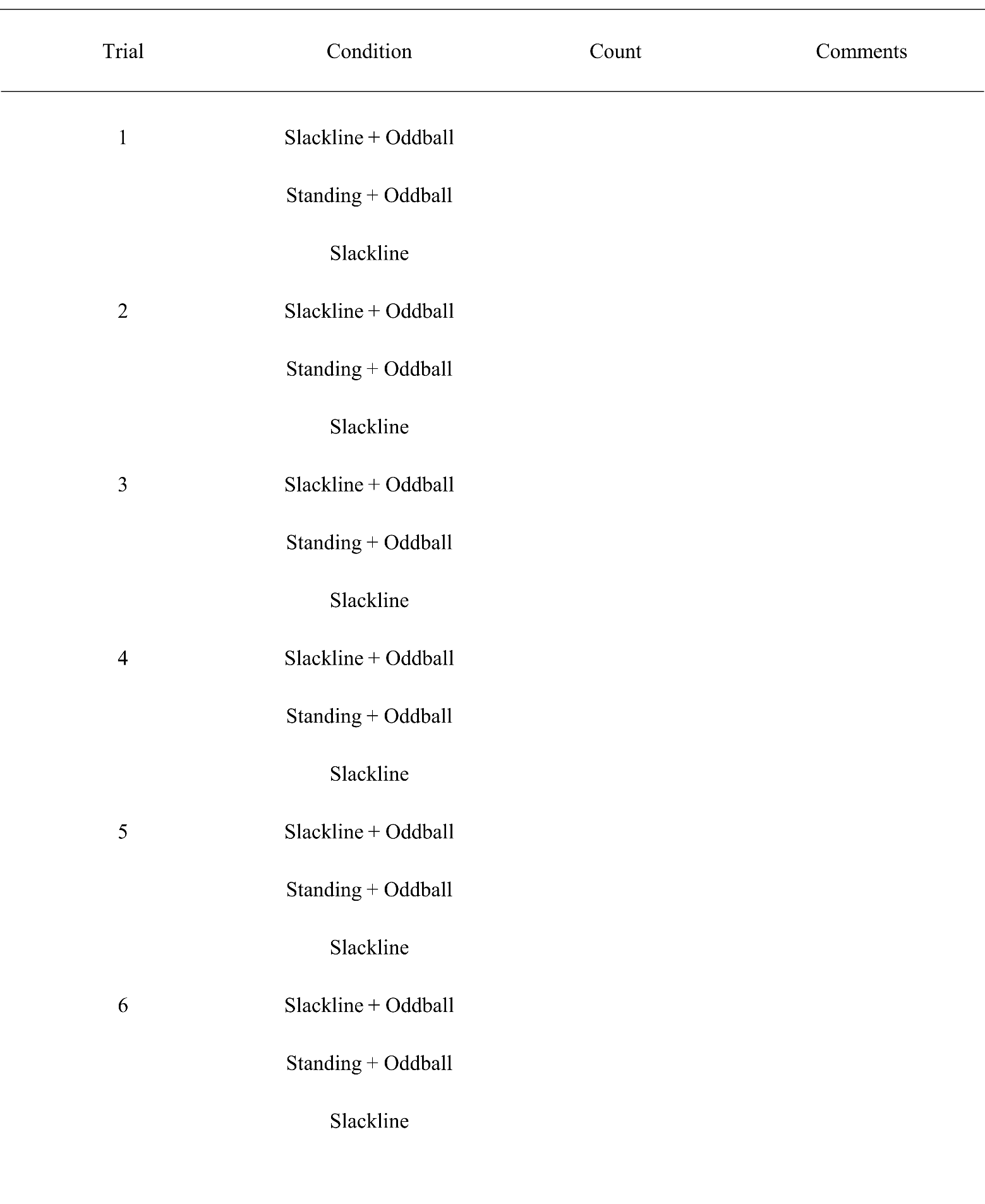


To assess participants' skill levels, we categorized them based on several criteria. Firstly, longest longline they successfully crossed without falling. Secondly, longest highline they successfully crossed. Thirdly, participants’ current length. According to participants’ response they were assigned to a skill level (see details in Supplementary Table 5). Additionally Supplementary Table 5 shows the number of participants assigned to a specific skill level.

**Supplementary Table 5**: Skill level definition and number of participants having a specific level


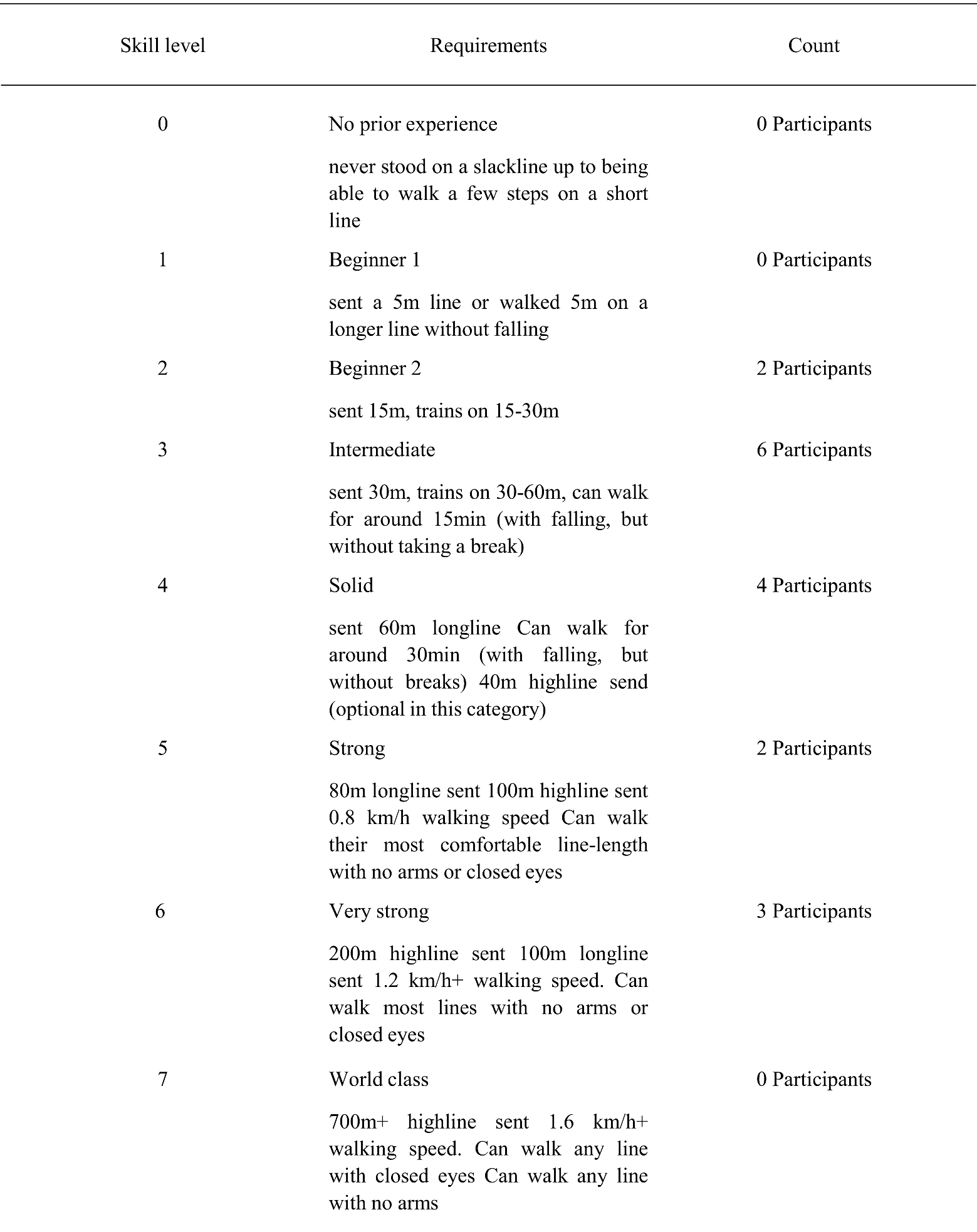

Supplement: Supplementary file 1 [file Data_Sheet_1.docx]
